# Nucleation of Huntingtin Aggregation Proceeds via Conformational Conversion of Pre‐Formed, Sparsely‐Populated Tetramers

**DOI:** 10.1002/advs.202309217

**Published:** 2024-03-12

**Authors:** Francesco Torricella, Vitali Tugarinov, G. Marius Clore

**Affiliations:** ^1^ Laboratory of Chemical Physics National Institute of Diabetes and Digestive and Kidney Diseases National Institutes of Health Bethesda MD 20892‐0520 USA

**Keywords:** elongation competent nuclei, huntingtin exon‐1 protein, NMR, pre‐nucleation tetramers, unified kinetic model of aggregation

## Abstract

Pathogenic huntingtin exon‐1 protein (htt^ex1^), characterized by an expanded polyglutamine tract located between the N‐terminal amphiphilic region and a C‐terminal polyproline‐rich domain, forms fibrils that accumulate in neuronal inclusion bodies, and is associated with a fatal, autosomal dominant neurodegenerative condition known as Huntington's disease. Here a complete kinetic model is described for aggregation/fibril formation of a htt^ex1^ construct with a 35‐residue polyglutamine repeat, htt^ex1^Q_35_. Using exchange NMR spectroscopy, it is previously shown that the reversible formation of a sparsely‐populated tetramer of the N‐terminal amphiphilic domain of htt^ex1^Q_35_, comprising a *D*_2_ symmetric four‐helix bundle, occurs on the microsecond time‐scale and is a prerequisite for subsequent nucleation and fibril formation on a time scale that is many orders of magnitude slower (hours). Here a unified kinetic model of htt^ex1^Q_35_ aggregation is developed in which fast, reversible tetramerization is directly linked to slow irreversible fibril formation via conversion of pre‐equilibrated tetrameric species to “active”, chain elongation‐capable nuclei by conformational re‐arrangement with a finite, monomer‐independent rate. The unified model permits global quantitative analysis of reversible tetramerization and irreversible fibril formation from a time series of ^1^H‐^15^N correlation spectra recorded during the course of htt^ex1^Q_35_ aggregation.

## Graphical abstract

A unified kinetic model of aggregation of the huntingtin exon‐1 protein, based on NMR data, is presented which directly links microsecond pre‐nucleation tetramerization with aggregation/fibril formation on the minutes/hours timescale via slow unimolecular conversion of excited states in the pool of sparsely‐populated tetramers into elongation‐competent “ground” state species.

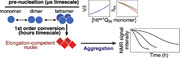

## 1 Introduction

The huntingtin htt^ex1^ protein, encoded by exon‐1 of the huntingtin gene *HTT*, comprises a 16‐residue N‐terminal amphiphilic domain (NT), a poly‐glutamine stretch (polyQ*_n_*) of variable length *n*, and a proline‐rich domain (PRD) containing two polyproline repeats.^[^ ^1^ ^]^ Extension of the polyQ region beyond ∼35 glutamines, by CAG expansion within exon‐1 of *HTT*, is responsible for Huntington's disease,^[^ ^1^, ^2^, ^3^ ^]^ a fatal neurodegenerative condition associated with htt^ex1^ aggregation and fibril accumulation within neuronal inclusion bodies.^[^ ^4^, ^5^, ^6^ ^]^ Solid‐state NMR and cryo‐electron microscopy studies have shown that htt^ex1^ fibrils consist of a central rigid β‐hairpin/β‐sheet polyQ core stabilized by a network of hydrogen bonds between glutamine side‐chains, with NT helices and highly mobile PRD domains located on the outside of the fibril.^[^ ^7^, ^8^, ^9^, ^10^, ^11^, ^12^, ^13^ ^]^ Quantitative exchange‐based solution NMR studies revealed that a very slowly aggregating htt^ex1^ construct with a 7‐residue glutamine repeat (htt^ex1^Q_7_), as well as a shorter construct (htt^NT^Q_7_) that lacks the PRD domain, undergo transient tetramerization of the NT region on the microsecond time scale,^[^ ^14^, ^15^, ^16^ ^]^ thereby increasing the local concentration of the polyQ tracts and providing a template for nucleation.

Reversible, microsecond time‐scale oligomerization generating sparsely‐populated tetramers of the htt^ex1^ NT domain, occurs *prior to* nucleation,^[^ ^15^ ^]^ but is critical for the formation of htt^ex1^ fibrils which occurs on a much slower time scale (minutes to hours),^[^ ^17^ ^]^ as evidenced by the fact that reduction of the tetramer population by a variety of mechanisms blocks fibril formation.^[^ ^15^, ^17^, ^18^ ^]^ In our earlier study of pre‐nucleation tetramerization and aggregation of a htt^ex1^ construct with a 35‐residue polyQ repeat, htt^ex1^Q_35_,^[^ ^17^ ^]^ we developed a set of tools for the quantitative analysis of both the kinetics of pre‐nucleation tetramerization and fibril formation from a series of 2D ^1^H‐^15^N band‐selective optimized flip‐angle short‐transient heteronuclear multiple quantum coherence (SOFAST‐HMQC)^[^ ^19^, ^20^ ^]^ NMR spectra. The kinetics of reversible tetramerization is derived from the concentration dependence of ^15^N/^1^H_N_ chemical shifts (δ_ex_) and ^1^H‐^15^N cross‐peak volume/intensity (V/I) ratios for residues located in the NT region of htt^ex1^Q_35_, while the kinetics of irreversible fibril formation is afforded by the decay of ^1^H‐^15^N cross‐peak intensities within the PRD domain, as the latter are not affected by the fast exchange processes (on the chemical shift time scale) associated with oligomerization.^[^ ^17^ ^]^

In our earlier study,^[^ ^17^ ^]^ the kinetic analysis of irreversible aggregation of htt^ex1^Q_35_ followed a conventional, “classical” approach,^[^ ^21^, ^22^ ^]^ with the rate of primary nucleation represented by an irreversible reaction of oligomerization of order 4. As a result, no direct connection was established in this treatment between fast, reversible tetramerization and the much slower, irreversible process of primary nucleation as, at the onset of aggregation, the concentration of tetramers exceeds that of nascent nuclei by 2‐to‐3 orders of magnitude.^[^ ^17^ ^]^ Here, we present a unified kinetic model of htt^ex1^Q_35_ aggregation that establishes a direct link between fast, reversible tetramerization and slow irreversible fibrillization. The unified model involves the conversion of pre‐formed tetramers to “active”, elongation‐capable nuclei by conformational re‐arrangement with a finite, monomer‐independent rate, and quantitatively accounts for the δ_ex_ and V/I data and aggregation profiles obtained from a series of SOFAST‐HMQC ^1^H‐^15^N correlation spectra acquired during the course of aggregation. The unified kinetic model presented here establishes a physically meaningful framework for the comprehensive, quantitative interpretation of htt^ex1^ aggregation data. Further, the combined analysis of the kinetics of fast, reversible htt^ex1^Q_35_ tetramerization and slow, irreversible aggregation provides new insights into the structural properties of htt^ex1^Q_35_ nuclei that may potentially be useful for inhibiting the aggregation process.

## 2 Results and Discussion

### 2.1 Unified Kinetic Model of htt^ex1^ Pre‐Nucleation and Fibril Formation

**Figure 1:**
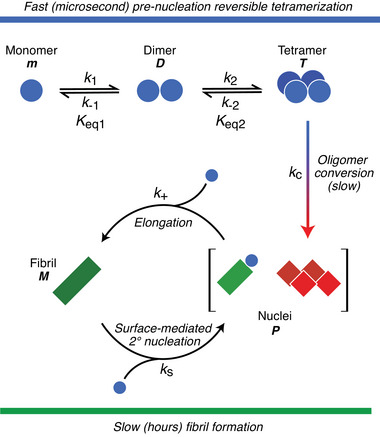
Schematic of the unified kinetic model of fast pre‐nucleation tetramerization coupled with slow monomer‐independent conversion, chain elongation and fibril surface‐mediated secondary nucleation steps of htt^ex1^Q_35_ fibrillization. *k*_1_ and *k*_2_ are association rate constants from monomer to dimer and from dimer to tetramer, respectively; *k*_−1_ and *k*_−2_ are the respective corresponding dissociation arte constants;    *K*_eq1_ and *K*_eq2_ are the dimerization and tetramerization equilibrium constants, respectively; *k*_c_ is the first order rate constant for the unimolecular conversion of pre‐nucleation transient tetramer *T* to nuclei *P*; and *k*_+_ and *k*_s_ are the rate constants for fibril elongation and surface‐mediated secondary nucleation, respectively. The equilibrium *m*⇌*D*⇌*T* is established “instantaneously”: *K*_eq,1_ = [*D*]/[*m*]^2^ = *k*_1_/*k*_‐1_; *K*_eq,2_ = [*T*]/[*D*]^2^ = *k*_2_/*k*_‐2_ and [*T*] = *K*_eq,2_[*D*]^2^ = *K*_eq,2_(*K*_eq,1_)^2^ = (*k*_2_/*k*_−2_)(*k*_1_/*k*_‐1_]^2^[*m*]^4^.

**Figure** **1** shows the unified kinetic scheme, in which reversible tetramerization is coupled to irreversible fibril formation. The monomers of htt^ex1^Q_35_ (*m*) interconvert on a timescale of ∼30 µs with sparsely populated dimeric species *D*, which can, in turn, interconvert with sparsely‐populated tetramers *T* on a timescale of < 100 µs. These two reversible oligomerization reactions are characterized by equilibrium constants *K*_eq,1_ and *K*_eq,2_, respectively (see Supporting Information). Note that a minor “off‐pathway” branch involving a non‐productive dimeric species that cannot further oligomerize,^[^ ^14^ ^]^ is present in all htt^ex1^ variants, but can be neglected for the purposes of this work^[^ ^16^, ^17^ ^]^ (and is therefore not shown in the scheme of Figure 1). The tetramer *T* is slowly and irreversibly converted (with a rate constant *k*_c_) in a monomer‐independent manner into elongation‐capable, “active” primary nuclei, followed by monomer‐dependent elongation (*k*_+_) to produce mature fibrils *M*. Surface‐mediated secondary nucleation^[^ ^23^, ^24^, ^25^ ^]^ (*k*_s_) involves interactions of htt^ex1^Q_35_ monomers *m* with fibrils *M* to produce secondary nuclei that, although physically distinct from the primary ones (i.e., converted tetramers), add to the total pool of nuclei characterized by the number concentration of extendable ends, *P* (Figure 1).

Analysis of the kinetics of oligomerization and fibril formation depicted in Figure 1, assumes that the equilibrium *m*⇌*D*⇌*T* is established “instantaneously”, and that the interconversion of oligomeric species at equilibrium occurs fast on the timescale of aggregation; that is the dependence of the concentration of tetramers *T* on the concentration of monomers *m*, follows the same law, *f*[*m*(*t*)], for each time‐point in the course of aggregation. The time course of aggregation can then be described by two coupled ordinary differential equations:^[^ ^17^, ^22^ ^]^

(1) $$\frac{dP}{dt}=k_{c}f\left[m\left(t\right)\right]+k_{s}m{\left(t\right)}^{n2}M\left(t\right)$$

(2) $$\frac{dM}{dt}=2k_{+}m\left(t\right)P\left(t\right)$$

where *f*[*m*(*t*)] is the time‐dependent concentration of tetramers; *P*(*t*), is the number concentration of extendable ends of the nascent fibrils (nuclei); *M*(*t*), is the mass concentration of mature fibrils (in monomer units); and the concentration of monomer is given by *m*(*t*) = *m*_tot_ – *M*(*t*), where *m*_tot_ is the total concentration of htt^ex1^Q_35_. *k*_c_ is the rate constant for the conversion of tetramers to “active” nuclei in units of h^−1^; *n*2, the order of secondary nucleation (in this instance *n*2 = 1); *k*_s_, the secondary nucleation rate constant in units of M^−^ *^n^* ^2^h^−1^; and *k*_+_, the chain elongation rate constant in units of M^−1^h^−1^. Assuming highly skewed fractional populations of the oligomeric states at equilibrium (i.e.*, p*_D_, *p*_T_ << *p*_m_), *f*[*m*(*t*)] can be expressed through the equilibrium/rate constants as:

(3) $$f[m(t)]={\left(K_{\mathrm{eq},1}\right)}^{2}K_{\mathrm{eq},2}m{\left(t\right)}^{4}={\left(\frac{k_{1}}{k_{-1}}\right)}^{2}\left(\frac{k_{2}}{k_{-2}}\right)m{\left(t\right)}^{4}$$

According to Equation (3), the first term on the right‐hand side of Equation (1), *k*_c_ *f*[*m*(*t*)], describing the monomer‐independent conversion of *all* pre‐equilibrated tetramers *T* to elongation‐capable tetramers via conformational re‐arrangement, is related to the conventional rate constant of primary nucleation of order 4 (i.e.*, k*_n_ *m*(*t*),^4^ where *k*_n_ is the primary nucleation rate constant) through the simple expression, *k*_n_ = (*k*_1_/*k*_‐1_)^2^(*k*_2_/*k*_‐2_)*k*_c_. The expressions for the rates of fibril‐mediated secondary nucleation (second term in Equation 1), as well as that of chain elongation (Equation 2), are not affected by the current model and remain the same as in our previous treatment.^[^ ^17^ ^]^

### 2.2 Global Fitting of the Experimental NMR Data

**Figure 2:**
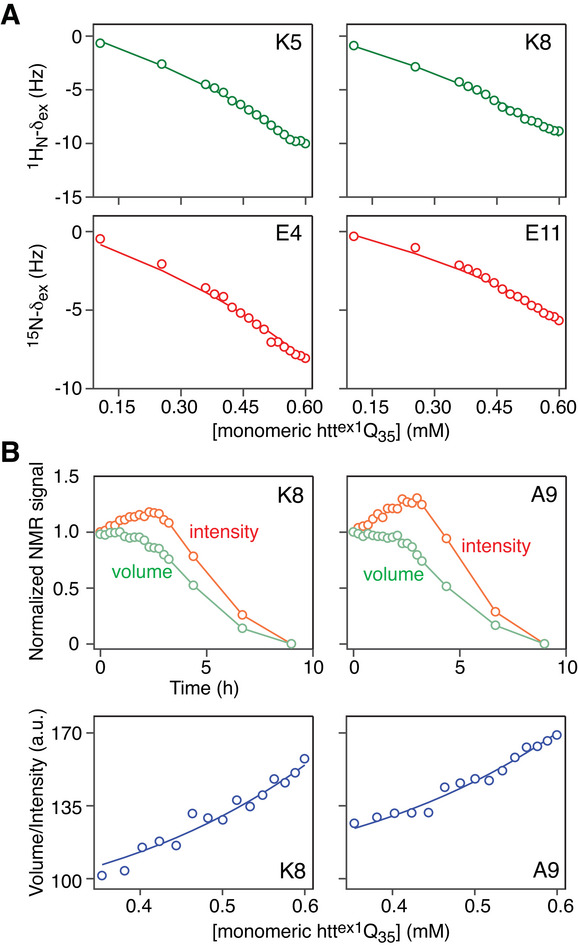
Pre‐nucleation tetramerization on the microsecond timescale. A) Concentration‐dependent ^1^H‐δ_ex_ (*top*) and ^15^N‐δ_ex_ (*bottom*) exchange‐induced chemical shifts. B) Time course of ^1^H‐^15^N cross‐peak volumes and intensities during aggregation (*top*) and ^1^H‐^15^N cross‐peak volume/intensity (*V*/*I*) ratios (*bottom*) as a function of the concentration of monomeric htt^ex1^Q_35_. The total sample concentration was 600 µm. The experimental data are shown as circles and the best fits to the unified kinetic scheme in Figure 1, under the assumption of equal changes in chemical shifts (Δ*ω*) between monomers and dimers and monomers and tetramers,^[^ ^14^, ^17^ ^]^ are shown as continuous solid lines. All experiments were recorded at 5 °C and 800 MHz (see the Experimental Section) and the data for other residues used in the analysis are provided in Figure S1 (Supporting Information). The optimized values of Δ*ω* are given in Table S1 (Supporting Information).

**Figure 3:**
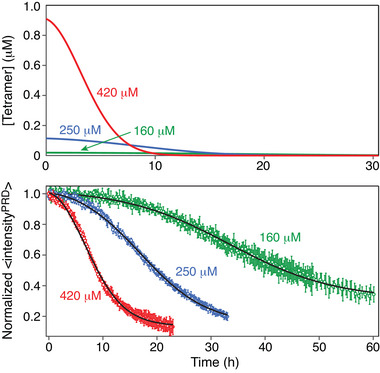
Quantitative analysis of htt^ex1^Q_35_ aggregation decay profiles. A) Simulated time‐dependence of the concentration of htt^ex1^Q_35_ tetramers *T* in monomer units for htt^ex1^Q_35_ samples with total monomer concentrations *m*_tot_ of 160, 250, and 420 µm calculated using the values of *K*_eq1_ and *K*_eq2_ determined from the data in Figure 1. B) Time‐dependence of the average ^1^H‐^15^N cross‐peak intensities for residues in the PRD domain of htt^ex1^Q_35_. The experimental data (shown as circles) were recorded at 5 °C and 800 MHz and normalized to the first time point (at *t* ≈ 0 h). The best‐fit curves are shown as black continuous lines and were obtained from a global fit to the kinetic scheme in Figure 1 and the model described by Equations (1) and (2) (see Experimental Section for details of the fitting procedure).

Our experimental NMR strategy follows closely that described in our previous study of htt^ex1^Q_35_ aggregation.^[^ ^17^ ^]^ The experimental data together with the global best fits for the unified kinetic model (Figure 1 and Equations (1), (2) and 3) to the concentration‐dependent ^15^N/^1^H_N_ δ_ex_ exchange‐induced shifts and *V*/*I* ratios and the decays in monomeric signal intensity (aggregation profiles) at three initial concentrations (160, 250, and 420 µm) of monomeric htt^ex1^Q_35_ obtained from the SOFAST ^1^H‐^15^N HMQC time series are shown in **Figures** **2** and **3** (Figure S1, Supporting Information), respectively (see the “Experimental Section” for details of the global fitting procedure and analytical methods used to calculate δ_ex_ and *V*/*I*). While NMR cross‐peak volumes are “immune” to chemical exchange line broadening associated with htt^ex1^Q_35_ oligomerization, the cross‐peak maximal intensities (heights; *I*) are affected by exchange line broadening. As a result, the volumes decrease monotonically with decreasing monomer concentration during the course of htt^ex1^Q_35_ aggregation, whereas the intensities of the cross‐peaks involved in exchange initially increase reaching a maximum, before decreasing (Figure 2B, *Upper Row*). This behavior is a direct consequence of chemical exchange line broadening associated with sub‐millisecond tetramerization. As the fractional population of the tetramer *T* has a cubic dependence on the concentration of the monomer *m*, exchange line broadening is reduced initially, and the concomitant increase of *I* due to line narrowing is larger than its decrease from the reduction in monomer concentration. The ratios of *V* and *I* (*V*/*I*), however, decrease monotonically with decreasing monomer concentration at the initial stages of aggregation (Figure 2B *, Lower Row*) following a simple analytical relationship (see “Experimental Section”).

The optimized values for *K*_eq,1_ and *K*_eq,2_ obtained from the global fit are 11.4 ± 0.3 and 5.6 (±0.5) x 10^4^ m ^−1^, corresponding to dimer and tetramer dissociation constants of ∼88 and ∼18 µm, respectively. The dissociation rate constants for the dimer (*k*_−1_) and tetramer (*k*_−2_) are ≥ 3 × 10^4^ and 2.4 (±0.5) x 10^4^ s^−1^, respectively (Figure S2, Supporting Information). At the highest concentration employed (600 µm), the fractional populations of dimer and tetramer in monomer units are ≈1.3 and ≈0.6%, respectively.

The temporal changes in the concentration of tetramers *T* in monomer units, 4[*T*] = 4*f*[*m*(*t*)], occurring during the course of aggregation for htt^ex1^Q_35_ samples with total htt^ex1^Q_35_ concentrations *m*_tot_ of 160, 250, and 420 µm, are shown in Figure 3A, while the corresponding htt^ex1^Q_35_ aggregation profiles best‐fit to the model depicted in Figure 1 and described by Equations (1) and (2), are shown in Figure 3B. Throughout this work, the decrease in the NMR signal arising from NMR visible htt^ex1^Q_35_ monomers as a function of time (Figure 3B) is attributed to the formation of any species (e.g., fibrils but also, if present, off‐pathway large oligomers)^[^ ^26^ ^]^ that are NMR invisible (unobservable) due to their very high molecular weight (see “Experimental Section”). The amount of tetrameric species available at the initial stages of fibril formation (early times in Figure 3A), defines the rate of primary nucleation (and, hence, the overall initial speed of aggregation) and is inversely proportional to the length of the lag period for the onset of aggregation (Figure 3B). At later times, the fibril‐mediated secondary nucleation process (*k*_s_) “takes over” and becomes the dominant mechanism for nucleation, thus rendering the amount of tetramers and the rate of their conversion to “active” nuclei largely inconsequential. We note that in the case of htt^ex1^Q_35_, the mechanism of secondary nucleation is likely to involve interactions of the polyQ region of the monomers with the polyQ tracts on the surface of mature fibrils.

**Figure 4:**
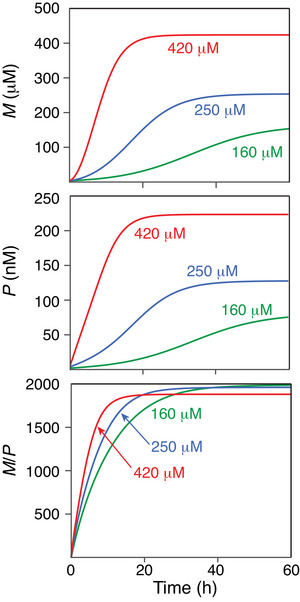
Simulation of the time dependence of mature fibrils *M*, nuclei *P*, and the *M*/*P* ratios during the course of htt^ex1^Q_35_ fibrillization at 5 °C using the optimized values of the rate constants (*k*_c_ = 0.07 ± 0.01 h^−1^; *k*_s_ = 0.3 ± 0.04 M^−1^h^−1^; and *k*_+_ = 6.4 (±0.6) x 10^5^ M^−1^h^−1^) obtained from the global fits to the experimental data shown in Figures 2 and 3.

Numerical integration of Equations (1) and (2) in the best‐fits of Figure 3B was performed using initial conditions, *P*(0) = 2, 4, and 6 nm for the 160, 250, and 420 µm samples of htt^ex1^Q_35_, respectively (each determined via a separate grid search), while *M*(0) was set to 0 for all samples (the fits with “seedless” initial conditions, *P*(0) = 0, were of significantly inferior quality), yielding the following optimal values of the rate constants: *k*_c_ = 0.07 ± 0.01 h^−1^; *k*_s_ = 0.3 ± 0.04 M^−1^h^−1^; and *k*_+_ = 6.4 (±0.6) x 10^5^ M^−1^h^−1^. The presence of a small amount of nuclei (*P*(0) ≠ 0) ensures partial de‐correlation^[^ ^22^, ^27^ ^]^ of otherwise highly correlated pairs of rate constants: (*k*_c_; *k*_+_) and (*k*_s_; *k*_+_) (see Supporting information). Plots of the time evolution of nuclei *P*, mature fibrils *M*, and the *M*/*P* ratio, which serves as an approximate measure of fibril length, are shown in **Figure** **4**. The ratio *M*/*P* reaches a maximum of ≈2000 at *t* → ∞ in all three cases, which corresponds to a fibril limiting length of ∼ 1 µm, assuming a 5 Å separation between polyQ β strands, in agreement with electron microscopy images in our earlier work.^[^ ^17^ ^]^ Of note, the best‐fits of htt^ex1^Q_35_ aggregation profiles to approximate analytical solutions of Equations (1) and (2) yield very similar values for the rate constants and practically identical quality of fit (see Supporting Information for the closed‐form solution of Equations (1) and (2)).

## 3 Discussion and Conclusion

In summary, we have described a kinetic model for aggregation of a pathogenic huntingtin construct with a 35‐residue polyglutamine repeat, htt^ex1^Q_35_. The model establishes a clear‐cut, quantitative connection between reversible tetramerization of htt^ex1^Q_35_, occurring on the microsecond timescale, and irreversible formation of fibrils that proceeds on a much slower timescale (from minutes to hours). The salient feature of this unified kinetic model involves a slow conversion step, in which the pre‐equilibrated pool of htt^ex1^Q_35_ tetramers is slowly converted with a finite, monomer‐independent rate to “active”, elongation‐capable nuclei via a process of conformational re‐arrangement of the polyQ tracts.

While there are some superficial similarities between the unified kinetic model of htt^ex1^Q_35_ aggregation described here and the recently developed kinetic model for aggregation of amyloid β42,^[^ ^28^, ^29^ ^]^ which involves reversible formation of oligomers that are converted in a monomer‐dependent manner to “active” nuclei, there are important differences between the two models that are specific to the system under consideration. First, the equilibrium *m*⇌*D*⇌*T* in Figure 1 is established “instantaneously” at each time‐point sampled during the course of aggregation decay, with transitions between monomers and tetramers occurring on the µs time‐scale – many orders of magnitude faster than the timescale of the nucleation and chain elongation processes. Second, only primary nucleation occurs via the conversion of tetramers in htt^ex1^Q_35_, while the process of secondary nucleation is modeled “classically” and likely involves interactions of the polyQ tracts of monomers with the same polyQ stretches ordered on the surface of mature fibrils. Third, the conversion stage is monomer‐independent (the order of the “conversion” reaction with respect to monomer *m* is 0) implying a conformational transition within the tetramer to generate an elongation‐capable species. The rate of conversion is determined by an unimolecular rate constant *k*_c_ that describes an inherently slow rate of “decay” of the “excited” states in the pool of sparsely‐populated htt^ex1^Q_35_ tetramers to “aggregation‐competent” (i.e., elongation‐capable) “ground state” species.

The slow conformational transition of the pool of pre‐nucleation tetramers to elongation‐active nuclei presumably involves the polyQ domain that eventually comprises the fibril core. In the pre‐nucleation tetramer, the polyQ domains are intrinsically disordered. The four‐helix bundle formed by the amphiphilic NT domain within the tetramer serves as a template to bring the polyQ domains of a pair of parallel‐oriented subunits into the appropriate spatial arrangement conducive to the formation of a four‐stranded β‐sheet comprising an intra‐subunit anti‐parallel β sheet/hairpin loop and an inter‐subunit parallel β‐sheet. This process is expected to be slow since a specific register between the polyQ strands is likely required to form elongation‐active nuclei. One may speculate that this process may be in part facilitated by interactions between the hydrophobic moieties of the glutamine sidechains, not dissimilar to the “hydrophobic zipper” model of protein folding.^[^ ^30^, ^31^ ^]^

We also note that the four‐helix NT bundle in the pre‐nucleation tetramer cannot be preserved in mature fibrils as these are characterized by a central rigid β‐hairpin/β‐sheet polyQ core with adjacent NT helices oriented parallel to one another.^[^ ^7^, ^8^, ^9^, ^10^, ^11^, ^12^, ^13^ ^]^ Thus the initial four‐helix NT bundle likely dissociates upon fibril elongation. However, the four‐helix NT bundle motif can be reformed upon intermolecular collision of fibril chains to generate branched fibrils.

As the NMR data described here were obtained at 5 °C, it is of interest to discuss our expectations for changes in htt^ex1^Q_35_ aggregation kinetics at higher, physiologically relevant temperatures. Although we do not expect the kinetic model as such to undergo any alterations at higher temperatures, the aggregation rate constants, as well as the equilibrium constants for htt^ex1^Q_35_ tetramerization are expected to change significantly. As shown by thioflavin T (ThT) fluorescence assays in our earlier study of the interaction of htt^ex1^Q_35_ with an SH3 domain,^[^ ^32^ ^]^ aggregation is accelerated at higher temperatures: the *t*_1/2_ for fibril formation of 100 µm htt^ex1^Q_35_ is ≈2 h as monitored by ThT fluorescence, compared to a *t*_1/2_ of ≈30 h for monomer disappearance at 5 °C as monitored by NMR. Thus, NMR‐based kinetic studies of htt^ex1^Q_35_ at 37 °C are not feasible. While the aggregation rate constants in Equations (1) and (2) (including that for tetramer conversion, *k*_c_), are expected to increase (possibly between 10‐ and 20‐fold) as the temperature is raised from 5 to 37 °C, it is more difficult to predict the temperature dependence of the equilibrium constants *K*_eq,1_ and *K*_eq,2_ (Equation 3), as lower intrinsic stabilities of dimers and tetramers (due to entropy losses associated with oligomer formation) may be counteracted by stronger hydrophobic effects at higher temperatures.

To place the unified kinetic model of htt^ex1^Q_35_ aggregation into a wider context, we note that the formation of non‐amyloid oligomeric species, so‐called “spherical oligomers”, has been reported in previous studies of htt^ex1^ aggregation.^[^ ^6^, ^33^ ^]^ In the framework of our model, such oligomers might be formed via the clustering of α‐helical tetramers into much larger aggregates that are nevertheless distinguishable from amyloid‐like fibrils. Although the possibility that “spherical oligomers” of htt^ex1^Q_35_ are indeed formed cannot be completely ruled out by kinetic modeling, the introduction of additional states/processes into the mechanism of htt^ex1^Q_35_ aggregation was not required to fit the NMR data in the present case or our previous work.^[^ ^17^ ^]^ The high‐quality best‐fits of htt^ex1^Q_35_ aggregation profiles obtained with the minimalistic kinetic model (Figure 3B and Equations (1) and (2)) fully satisfy the experimental data, and are devoid of any systematic deviations that would serve as an indication of the presence of additional processes not included in the model. In contrast, in our recent NMR‐based study of aggregation kinetics of Aβ42,^[^ ^26^ ^]^ the introduction of an additional step for “off‐pathway” oligomer formation to an otherwise similar kinetic model, was critically important to best fit the aggregation profiles quantitatively. We therefore conclude that further complication of the mechanism of htt^ex1^Q_35_ aggregation (i.e., the incorporation of additional processes) is not warranted by our experimental data.

The unified quantitative kinetic model of htt^ex1^Q_35_ aggregation described here bears a superficial similarity to the qualitative (cartoon‐like) model of htt^ex1^ aggregation proposed by Wetzel and co‐workers^[^ ^6^, ^33^, ^34^ ^]^ (see, for example, Figure 3 of ref. [6]). The common feature of these models is the reversible formation of htt^ex1^ tetrameric species as the first step in the nucleation process. While in our model, the tetramers undergo an irreversible transition to form elongation‐capable nuclei, the mechanism of nucleation posited by Wetzel includes an additional reversible step that involves clustering of tetramers into α‐helical higher‐order oligomers (see discussion above) that are, in turn, reversibly converted to kinetic nuclei (elongation‐capable species) with increased local concentrations of polyQ chains. In this regard, it is important to stress that the qualitative model proposed by Wetzel is not based on detailed global fitting of quantitative kinetic data at multiple htt^ex1^ concentrations, and our current results provide no evidence for the existence of higher‐order (>4) oligomers that are subsequently converted into elongation‐competent species.

Finally, it is worth noting that the analytical framework described in the paper will allow us to interpret in a physically meaningful, quantitative manner htt^ex1^Q_35_ aggregation profiles as a function of a variety of experimental conditions (for example, in the presence of crowding agents, macromolecular co‐solutes, different salt concentrations, etc.) that we are currently studying in our laboratory. Note that different solution conditions can have an impact on any or all of the stages of the htt^ex1^Q_35_ aggregation mechanism. In the absence of a kinetic model that unifies all the involved processes, the monitored changes in aggregation would be largely uninterpretable.

## 4 Experimental Section

### Expression and Purification of htt^ex1^Q_35_

htt^ex1^Q_35_ was expressed as a fusion protein with the immunoglobulin‐binding domain of streptococcal protein G, GB1, attached to the N‐terminal end and separated by a factor Xa cleavage site as described previously.^[^ ^17^, ^32^ ^]^ Uniform ^15^N‐labeling and cleavage by factor Xa to remove GB1 were carried out as described previously,^[^ ^15^, ^17^ ^]^ except that dialysis was performed against buffer containing 50 m Tris‐HCl (pH 8.0), 100 mm NaCl and 1.5 mm dithiothreitol (DTT) using a 3.5 kDa cut‐off dialysis membrane. The presence of significant amounts of DTT prevents the oxidation of the Met^7^ side‐chain to a sulfoxide. Following the last purification step,^[^ ^17^ ^]^ the lyophilized htt^ex1^Q_35_ fractions were dissolved in a 1:1 (v/v) mixture of trifluoracetic acid (TFA) and hexafluoroisopropanol (HFIP) ensuring complete removal of pre‐existing aggregates.^[^ ^17^, ^35^ ^]^ Protein identity and completion of the cleavage reaction were verified by liquid phase chromatography coupled with electrospray ionization mass spectrometry.

### NMR Sample Preparation

All NMR samples of htt^ex1^Q_35_ were prepared by first dissolving an aliquot of purified protein in a 13.8 mm monobasic sodium phosphate buffer, pH 4.6, containing 50 mm NaCl and 10% D_2_O/90% H_2_O (v/v). Upon re‐suspension, the protein solution was centrifuged at 20 000 g for 25 min. to remove any pre‐formed aggregates. The pH of the buffer was subsequently adjusted to 6.5 by adding dibasic sodium phosphate for a final sodium phosphate concentration of 20 mm. Protein concentrations were determined by UV absorbance at 205 nm.^[^ ^36^ ^]^ The samples were placed in 5 mm Shigemi tubes.

### NMR Spectroscopy

All NMR experiments were recorded at 5 °C using a Bruker 800 MHz Avance‐III spectrometer, equipped with a TCI triple resonance *x*,*y*,*z*‐axis gradient cryogenic probe. NMR data were processed using the NMRPipe/NMRDraw software suite^[^ ^37^ ^]^ and analyzed with the program Sparky.^[^ ^38^ ^]^ Aggregation of ^15^N‐labeled htt^ex1^Q_35_ was monitored by following ^1^H‐^15^N cross‐peak intensities (heights) and volumes from a series of 2D ^1^H‐^15^N SOFAST‐HMQC spectra.^[^ ^19^, ^20^ ^]^ Each 2D NMR spectrum was acquired using a recycle delay of 0.3 s and a total of 100* x 2048* complex data points in the indirect (^15^N) and direct (^1^H) dimensions with respective acquisition times of 44 and 104 ms. The number of scans for each *t*_1_ increment was set to 16 resulting in total acquisition time of ≈11 min per 2D spectrum.

### Measurement of Concentration Dependent ^15^N/^1^H_N_ Exchange‐Induced Chemical Shifts (δ_ex_)

Concentration dependent changes in ^15^N/^1^H_N_ chemical shifts were measured on a 600 µm ^15^N‐labeled sample of htt^ex1^Q_35_ from a series of 2D ^1^H‐^15^N SOFAST‐HMQC spectra. The concentration of monomeric htt^ex1^Q_35_ as a function of time, *m*(*t*), was recast from the average normalized cross‐peak intensity of 11 residues of the htt^ex1^Q_35_ PRD domain (<*I*_PRD_>) as, *m*(*t*) = *m_tot_* [<*I*_PRD_(*t*)> − <*I*_PRD_(∞)>]/[<*I*_PRD_(0)> − <*I*_PRD_(∞)>]. For each time‐point *i*, ^15^N/^1^H_N_‐δ_ex_ values were calculated as, $\delta_{\mathrm{ex}}\left(i\right)=\delta_{\mathrm{obs}}\left(i\right)-\delta_{\mathrm{obs}}^{\mathrm{ref}}$, where δ_obs_(*i*) is the observed chemical shift at the monomer concentration *m*(*i*), and $\delta_{\mathrm{obs}}^{\mathrm{ref}}$ is the chemical shift at the concentration close to the end of the aggregation decay (≈20 µm).

### Global analysis of the concentration dependence of exchange‐induced chemical shifts and V/I ratios together with the aggregation profiles of htt^ex1^Q_35_

Combined analysis of ^15^N/^1^H_N_‐δ_ex_, ^1^H^N^‐^15^N cross‐peak volume/intensity (V/I) ratios and aggregation profiles of htt^ex1^Q_35_ followed the procedures described previously.^[^ ^17^ ^]^ The experimental concentration dependence of ^15^N‐δ_ex_, ^1^H_N_‐δ_ex_, ^1^H‐^15^N cross peak V/I ratios, and decay profiles of averaged normalized NMR signal intensities, I, were fit simultaneously by the minimization of the error function,

(4)

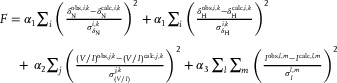

where the first and second terms correspond to the differences between the observed (“obs”) and calculated (“calc”) ^15^N‐δ_ex_ and ^1^H_N_‐δ_ex_ values, respectively, for residue *i*, measured at each concentration *k* of the monomer; the third term accounts for the differences between observed and calculated *V/I* ratios for residue *j*, measured at each monomer concentration *k*; and the last term describes the differences in observed and calculated averaged normalized NMR signal intensities, *I*, measured over all time‐points *l* at the total protein concentration *m* for the 11 PRD residues of htt^ex1^Q_35_; *σ* are experimental errors (assumed to be approximately equal to one‐half of the standard deviation of the distribution of peak intensities *I* in the aggregation profiles); and the coefficients *α*_1_, *α*_2_ and *α*_3_ are empirically determined weighting factors.

The following form of the NMR Liouvillian for the three‐state exchanging system, $m\underset{k_{-1}}{\overset{k_{1}^{\mathrm{app}}}{⇌}}D\underset{k_{-2}}{\overset{k_{2}^{\mathrm{app}}}{⇌}}T$ was used for all calculations of ^15^N/^1^H_N_‐δ_ex_ values and *V*/*I* ratios: $\tilde{R}={\tilde{R}}^{cs}+{\tilde{R}}^{r}+{\tilde{R}}^{ex}$, where ${\tilde{R}}^{cs}$, ${\tilde{R}}^{r}$ and ${\tilde{R}}^{ex}$ are the chemical shift, intrinsic transverse relaxation and chemical exchange contributions, respectively, given by: ${\tilde{R}}^{cs}$ = *diag*[0; ‐*i*Δ*ω_n_*; ‐*i*Δ*ω_n_*] for single‐quantum coherences (see Supporting Information of ref. [17] for the form of ${\tilde{R}}^{cs}$ used for the zero‐ and double‐quantum magnetization in calculations of *V*/*I* ratios), where Δ*ω_n_* is the difference between the chemical shifts of the monomer and dimer/tetramer (assumed the same for dimers and tetramers; rad/s) for nucleus *n* (^15^N or ^1^H_N_); ${\tilde{R}}^{r}$ = ‐*diag*[*R*_2,_ *_n_*; 2*R*_2,_ *_n_*; 4*R*_2,_ *_n_*], where *R*_2,_ *_n_* is the transverse spin relaxation rate of the monomer for nucleus *n* in the absence of exchange (s^−1^); and the “linearized” form of exchange matrix ${\tilde{R}}^{ex}$ is given by,

(5) $${\tilde{R}}^{ex}=-\left(\begin{array}{ccc} k_{1}^{\mathrm{app}} & -k_{-1} & 0\\ -k_{1}^{\mathrm{app}} & k_{2}^{\mathrm{app}}+k_{-1} & -k_{-2}\\ 0 & -k_{2}^{\mathrm{app}} & k_{-2} \end{array}\right)$$

where $k_{1}^{\mathrm{app}}$ and $k_{2}^{\mathrm{app}}$ are apparent, pseudo‐first order rate constants given by, 2*k*_1_[*m*] and 2*k*_1_ *k*_2_[*m*]^2^/*k*_‐1_, respectively.^[^ ^17^ ^]^ The values of δ_ex_ were calculated from the *imaginary* part of the smallest (by absolute magnitude) eigenvalue of the matrix $\tilde{R}$: δ_ex_(Hz) = *Im*(min[*eig*{$\tilde{R}$})/2π, where *eig*{$\tilde{R}$} is a vector of complex eigenvalues of $\tilde{R}$. The ratios *V*/*I* were calculated from the approximate relationship described previously,^[^ ^17^ ^]^ (see Supporting Information of ref. [17] for details) with the “effective” relaxation rates, *R*_2,eff_ = *R*_2_ + *R*_ex_, estimated from the *real* part of the smallest (by absolute magnitude) eigenvalue of the matrix $\tilde{R}$: *R*_2,eff_ = ‐*Re*(min[*eig*{$\tilde{R}$}). Best‐fit concentration‐dependent amide backbone ^15^N/^1^H_N_ exchange‐induced shifts and *V*/*I* ratios for the residues of htt^ex1^Q_35_ NT domain not included in Figure 2 of the main text are shown in Figure S1 (Supporting Information).

The Δ*ω*_N_ values were set to those previously determined for htt^NT^Q_7_^[^ ^14^ ^]^ on the assumption that the structure of the helical coiled‐coil tetramer formed by the NT domain is not affected by the length of the polyQ tract, while the Δ*ω*_H_ values were optimized with the starting values reported in the previous study,^[^ ^17^ ^]^ and using constrained minimization with the upper and lower bounds set to 2 times the uncertainties in the reported values (Table S1, Supporting Information). Intrinsic (exchange‐free) ^15^N‐*R*_2_ and ^1^H_N_‐*R*_2_ relaxation rates for htt^ex1^Q_35_ monomer were taken from the previous study.^[^ ^17^ ^]^ Since concentration‐dependent ^15^N‐*R*_1ρ_ measurements are not feasible on a rapidly aggregating system,^[^ ^16^ ^]^ the approximate range of *k*_‐1_ values was established using a grid search (Figure S2, Supporting Information), and subsequently fixed at a value of 30 000 s^−1^.

It is worth noting that if one uses the approximation for ^15^N‐δ_ex_ valid in the fast‐exchange regime on the chemical shift time scale and given by, δ_ex_ ≈ (*p*_D_ + *p*_T_)Δ*ω* = [2*K*_eq,1_ *m* + 4(*K*_eq,1_)^2^ *K*_eq,2_ *m* ^3^]Δ*ω*,  both *K*_eq,1_ and *K*_eq,2_ are underestimated by only ∼2.5%; using the same approximation for both ^15^N‐δ_ex_ and ^1^H_N_‐δ_ex_, however, results in overestimation of *K*_eq,1_ and underestimation of *K*_eq,2_ by ∼2.5% and ∼20%, respectively.

Temporal changes in NMR signal intensities *I* monitored in aggregation profiles of htt^ex1^Q_35_ were calculated from the expression,

(6) $$I_{\mathrm{calc}}=A_{0}\left[1-\alpha^{\prime }M\left(t\right)/m_{tot}\right]$$

where *α*′ is a scaling factor that takes into account that NMR intensities for the PRD domain do not decay to exactly zero for fully aggregated samples (as the PRD remains flexible and disordered in the fibrils); *A*_0_ is an overall scaling factor that accounts for possible errors in normalization of NMR intensities; and *M*(*t*) is the mass concentration of mature fibrils derived from numerical integration of Equations (1) and (2) . Numerical integration of Equations (1) and (2)  was performed using an explicit Runge–Kutta (1,4) formula implemented in Matlab “ode45” solver for non‐stiff ordinary differential equations,^[^ ^39^ ^]^ with integration steps corresponding to the time‐points sampled in the NMR experiments.

The set of global variable parameters in the minimization of the target function thus comprised: {*K*_eq,1_; *K*_eq,2_; *k*_‐2_; *k*_c_; *k*_s_; *k*_+_}. Of note, although a single (combined) target function (Equation 4) was used in the global analysis of the experimental data, the first three terms in Equation (4) drive the determination of *K*_eq,1_ and *K*_eq,2_, since the aggregation profiles are tolerant to the “supplied” values of *K*_eq,1_ and *K*_eq,2_ by virtue of the dependence of the rate of production of nuclei, *dP*/*dt* in Equation (1), on the product of the tetramer concentration, *f*[*m*(*t*)], and the conversion rate constant *k*_c_ in the model. For *k*_‐1_ set to 3 × 10^4^ s^−1^, *k*_‐2_ optimizes to 2.4 (± 0.5) x 10^4^ s^−1^.

The uncertainties in the values of the optimized parameters, corresponding to confidence intervals of ±1 standard deviation, were determined from the variance‐covariance matrix of the nonlinear fit. Uncertainties in the rate constants recalculated from the optimized parameters (*k*_1_ and *k*_2_) were determined by standard error propagation. All calculations were performed using an in‐house program written in MATLAB (MathWorks Inc, MA).

## Conflict of Interest

The authors declare no conflict of interest.

## Supporting information

Supporting Information

## Data Availability

The data that support the findings of this study are available in the Supporting Information of this article.
