# Supplementary material for: Nucleation of Huntingtin Aggregation Proceeds via Conformational Conversion of Pre‐Formed, Sparsely‐Populated Tetramers
Source: Adv Sci (Weinh). 2024 Mar 12;11(24):2309217. doi: 10.1002/advs.202309217 (PMC11199967; doi:10.1002/advs.202309217)
Supplement: Supplementary file 1 — Supporting Information [file ADVS-11-2309217-s001.pdf]

ADVANCED SCIENCE
Open Access

Supporting Information

for *Adv. Sci.*, DOI 10.1002/advs.202309217

Nucleation of Huntingtin Aggregation Proceeds via Conformational Conversion of Pre-Formed, Sparsely-Populated Tetramers

Francesco Torricella, Vitali Tugarinov and G. Marius Clore**

Supplementary Information

Nucleation of Huntingtin Aggregation Proceeds Via Conformational Conversion of Pre-formed, Sparsely-Populated Tetramers

Francesco Torricella, Vitali Tugarinov* and G. Marius Clore*

Laboratory of Chemical Physics, National Institute of Diabetes and Digestive and Kidney Diseases, National Institutes of Health, Bethesda, MD 20892-0520.

- 1 Mathematical Appendix
- 1 Supplementary Table
- 2 Supplementary figures

*To whom correspondence should be addressed: G.M.C., mariusc@mail.nih.gov and V.T., vitali.tugarinov@nih.gov

Appendix

Closed-form, approximate analytical solutions of Eqs. (1) and (2) for *n*2 = 1 and initial conditions, {*P*(0) = P_0; *M*(0) = 0}. It is possible to obtain a closed-form analytical solution of Eqs. (1) and (2) for *n*2 = 1 and initial conditions, {$P(0) = P_0$; $M(0) = 0$}, using the 'fixed-point' iteration scheme,[1-3]

$$\frac{M(t)}{m_{tot}} = 1 - \left[\frac{(B_+ + C_+)(B_- + C_+ e^{\kappa t})}{(B_- + C_+)(B_+ + C_+ e^{\kappa t})}\right]^{\frac{k_\infty^2}{\kappa \bar{k}_\infty}} e^{-k_\infty t} \tag{S1.1}$$

where

$$\kappa = m_{tot}\sqrt{2k_+ k_s} \tag{S1.2}$$

$$\lambda = K_{eq,1} m_{tot}^2 \sqrt{2K_{eq,2} k_+ k_c} \tag{S1.3}$$

$$C_\pm = \frac{k_+}{\kappa} P_0 \pm \frac{\lambda^2}{2\kappa^2} \tag{S1.4}$$

$$k_\infty = \sqrt{(2k_+ P_0)^2 + K_{eq,2}(K_{eq,1})^2 k_+ k_c m_{tot}^4 + 2k_+ k_s m_{tot}^2} \tag{S1.5}$$

$$\bar{k}_\infty = \sqrt{k_\infty^2 - 4C_+ C_- \kappa^2} \tag{S1.6}$$

$$B_\pm = \frac{k_\infty \pm \bar{k}_\infty}{2\kappa} \tag{S1.7}$$

and the same notation is used as in the main text. It follows from Eqs. (S1) that for $P_0 = 0$ ('seedless' onset of aggregation), the pairs of rate constants, (k_c; k_+) and (k_s; k_+), are correlated, and, hence, only the products ($k_c k_+$) and ($k_s k_+$) can be determined with confidence. The aggregation profiles for htt^{ex1}Q$_{35}$ were best-fit to Eqs. (S1) using initial conditions, *P*(0) = 1.6, 3.2, and 4.8 nM for 160, 250 and 420 μM samples of htt^{ex1}Q$_{35}$, respectively (*i.e.* 20 % lower than *P*(0) values used in the calculations with numerical integration in the main text), with *M*(0) set to 0 for all profiles. These fits yielded the following values for the rate constants: $k_c = 0.08 \pm 0.01$ hr^{1}; $k_s = 0.3 \pm 0.04$ M^{-1}h^{1}; and $k_+ = 6.5$ (± 0.8) x 10^5 M^{-1}h^{-1}, which compare remarkably well with those reported in the main text. The quality of the fit was indistinguishable from the one with numerical integration of Eqs. (1), with the average *M*/*P* ratio at $t \rightarrow \infty$ over the three analyzed profiles, <*M*/*P*> ~ 2,000.

Table S1. Residue specific values for the differences in ^{15}N and $^{1}H_N$ $\Delta\omega$ chemical shifts between the dimer/tetramer (*D*/*T*) and the monomer *m* for the NT residues used in the global fitting.[a]

Residue	$\Delta\omega_{D/T}$ (ppm)
**^{15}N**	
4	-5.52 ± 0.96
5	-3.04 ± 0.56
8	-3.00 ± 0.62
9	-2.00 ± 0.48
11	-3.95 ± 0.74
**$^{1}H_N$**	
5	-0.77 ± 0.01
6	-0.21 ± 0.01
8	-0.64 ± 0.01
9	-0.56 ± 0.01
12	-0.26 ± 0.01
13	-0.60 ± 0.01

[a]The values of ^{15}N $\Delta\omega_{D/T}$ were taken from Kotler et al.[4] while those for $^{1}H_N$ $\Delta\omega_{D/T}$ were optimized.

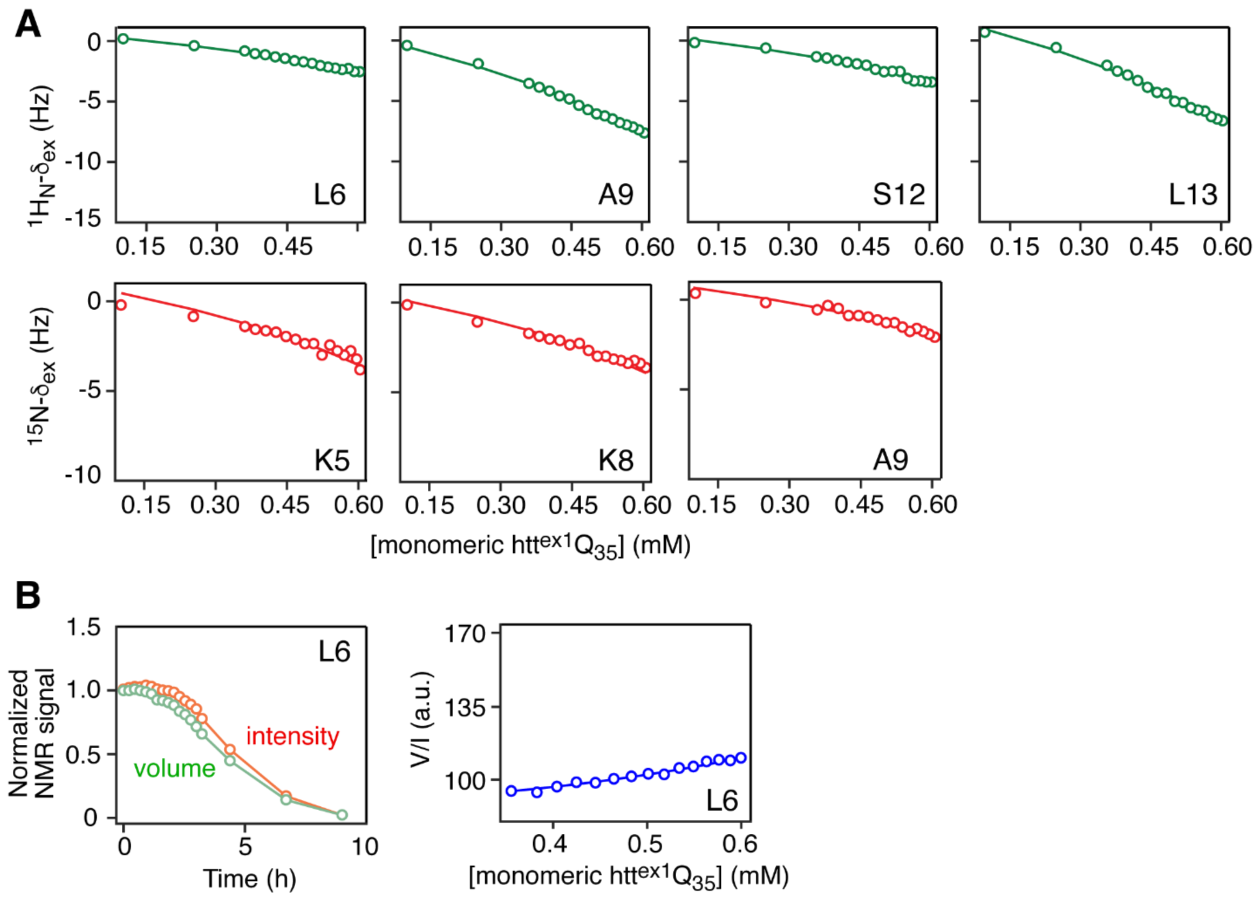

Figure S1. Concentration-dependent $^{1}H_{N}/^{15}N$ exchange-induced chemical shifts and volume/intensity ratios. (A) Concentration-dependent amide backbone $^{1}H_{N}$ exchange-induced chemical shifts (^{1}H-δ_{ex}; *top panels*) and their ^{15}N counterparts (^{15}N-δ_{ex}; *bottom panels*) for the residues of the NT domain of htt^{ex1}Q$_{35}$ not included in Fig. 2 of the main text. ^{1}H-^{15}N cross-peak volume/intensity ratio versus vs. time of htt^{ex1}Q$_{35}$ aggregation (*left panel*), and concentration dependence of the ^{1}H-^{15}N cross-peak volume/intensity ratios(V/I) are for Leu^{6} (*right panel*). The total sample concentration was 600 μM. The experimental data are shown as circles and the best fits to the tetramerization scheme are represented by continuous solid lines. All experiments were recorded at 5 °C and 800 MHz.

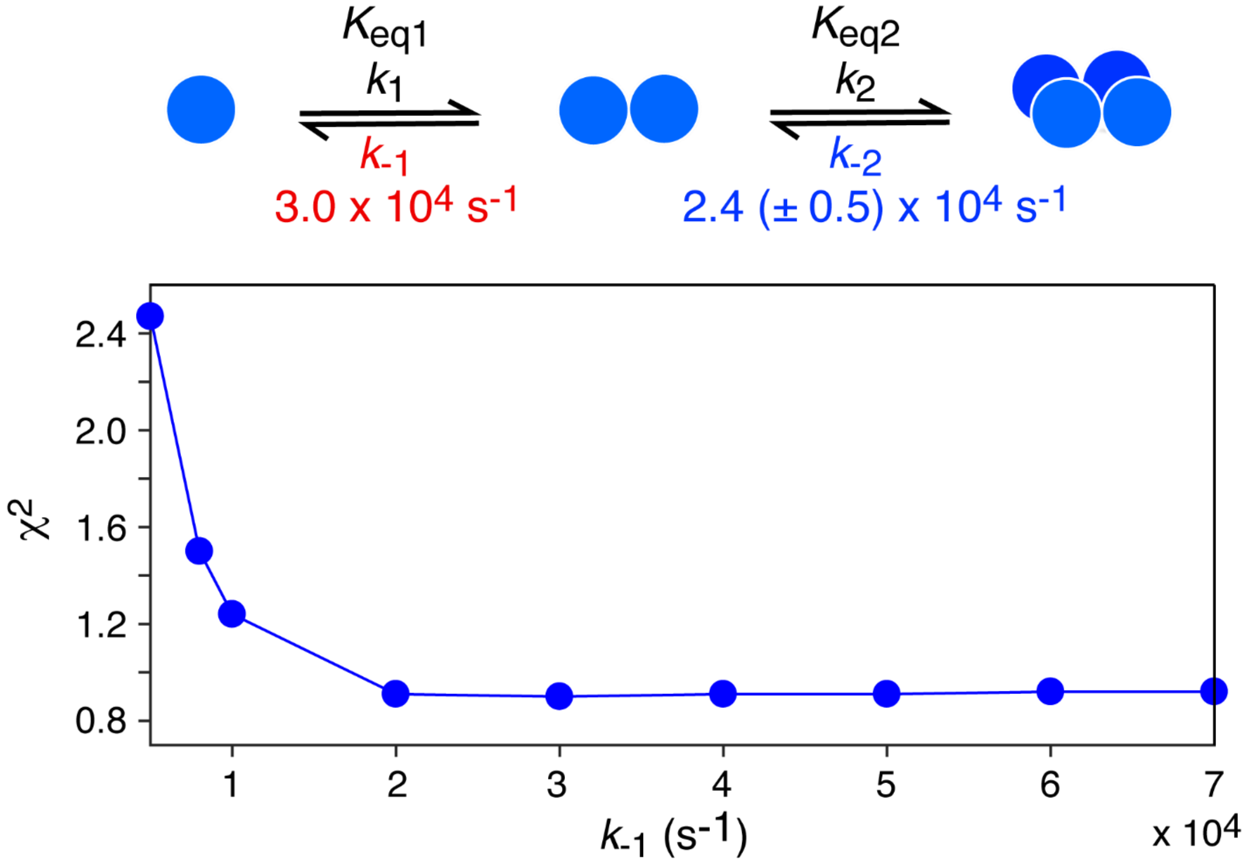

Figure S2. Grid search of χ^2 versus k_{-1} (the rate constant for dissociation of the dimer *D*) obtained from the global fits to the concentration dependence of the $^{15}N/^{1}H_N$ exchange induced shifts and volume/intensity ^{1}H-^{15}N cross-peak ratios shown in Fig. 2 of the main text. The equilibrium constants $K_{eq,1}$ and $K_{eq,2}$, as well as the rate constant for dissociation of the tetramer *T*, k_{-2}, are optimized in the search.

Supplementary References

[1] T. P. Knowles, C. A. Waudby, G. L. Devlin, S. I. Cohen, A. Aguzzi, M. Vendruscolo, E. M. Terentjev, M. E. Welland, C. M. Dobson, *Science* **2009**, *326*, 1533-1537.
[2] S. I. Cohen, M. Vendruscolo, C. M. Dobson, T. P. Knowles, *J Chem Phys* **2011**, *135*, 065106.
[3] S. I. Cohen, S. Linse, L. M. Luheshi, E. Hellstrand, D. A. White, L. Rajah, D. E. Otzen, M. Vendruscolo, C. M. Dobson, T. P. Knowles, *Proc Natl Acad Sci U S A* **2013**, *110*, 9758-9763.
[4] S. A. Kotler, V. Tugarinov, T. Schmidt, A. Ceccon, D. S. Libich, R. Ghirlando, C. D. Schwieters, G. M. Clore, *Proc Natl Acad Sci U S A* **2019**, *116*, 3562-3571.
